# Agarose Hydrogels Enriched by Humic Acids as a Functional Model for the Transport of Pharmaceuticals in Nature Systems

**DOI:** 10.3390/molecules29245937

**Published:** 2024-12-16

**Authors:** Martina Klučáková, Petra Závodská

**Affiliations:** Faculty of Chemistry, Brno University of Technology, Purkyňova 118/464, 612 00 Brno, Czech Republic; enev@fch.vutbr.cz

**Keywords:** hydrogel, humic acid, transport, sulphapyridine, diclofenac

## Abstract

The presence of pharmaceuticals in nature systems poses a threat to the environment, plants, animals, and, last but not least, human health. Their transport in soils, waters, and sediments plays important roles in the toxicity and bioavailability of pharmaceuticals. The mobility of pharmaceuticals can be affected by their interactions with organic matter and other soil and water constituents. In this study, a model agarose hydrogel enriched by humic acid as a representative of organic matter is used as a transport medium for pharmaceuticals. Sulphapyridine (as a representative of sulphonamide antibiotics) and diclofenac (as a representative of widely used non-steroidal anti-inflammatory drugs) were chosen for experiments in diffusion cells. Pharmaceuticals were passed through the hydrogel from the donor solution to the acceptor compartment and could interact with humic acids incorporated in the hydrogel. The lag time was prolonged if the hydrogel was enriched by humic acids from 134 to 390 s for sulphapyridine and from 323 to 606 s for diclofenac. Similarly, the incorporation of humic acids in the hydrogel resulted in a decrease in the determined diffusion coefficients. The decrease was stronger in the first stage of the experiment when diffusing particles could interact with vacant binding sites.

## 1. Introduction

Agarose hydrogels are widely used in many practical applications. They are widely used materials due to their inexpensive, non-toxic, easy synthesis, and good experimental properties. Their well-known uses are the separation in column chromatography and bacterial culture support [1,2]. Many studies are focused on the hydrogel internal structure [3,4,5] and gelling behaviour [6,7,8,9]. Nevertheless, relationships between the molecular structure of agarose, hydrogel micro-scale structural factors, and the transport of different particles in agarose hydrogels are still a matter of debate [3,4,9]. Agarose hydrogels are widely used as a transport medium for the determination of diffusion characteristics of different substances [2,10,11,12,13,14]. Some authors reported that the main path for diffusing particles is relatively large pores (filled with water) between agarose chains [3,15,16,17,18]. Favourable transport properties of agarose hydrogels are widely utilized passive samplers based on diffusion gradients in thin hydrogel films [15,16,19]. Other authors studied the diffusion of different substances in agarose hydrogels and determined diffusion coefficients for proteins [10,20,21], enzymes [22], pharmaceuticals [23,24], metal ions [12,15,25], dyes [12,26,27], dextran [10], and DNA [10].

In this work, an agarose hydrogel was enriched by humic acids as an active substance able to interact with diffusion particles. Humic acids were incorporated into the hydrogel to investigate their effect on the transport of pharmaceuticals. Thus, hydrogels containing a characteristic content of humic acids can be considered as a model of natural systems. It is known that humic substances can occur in nature in different forms: as insoluble in a suspended state, in the form of a colloidal humic sol, and in the form of swollen hydrogels, which inspired us to investigate the transport of pharmaceuticals in hydrogels. Another reason was that it is easy to prepare hydrogel samples with a defined size and shape, which is important for the mathematical processing of experimental data.

Pharmaceuticals are indispensable in modern human and veterinary medicine to secure the health of a growing world population and consumed livestock. They can occur in nature as a result of manufacturing processes, the disposal of unused products, and animal excretion [28,29,30]. Therefore, they can be detected in natural waters, sediments, and soils and are recognized as emerging environmental contaminants [31,32,33,34,35,36]. They can undergo sorption on natural soil and water components, degradation, and chemical transformation into structurally similar compounds [37,38,39,40]. The fate and behaviour of pharmaceuticals and their residues in soil can be effectively affected by their interactions with soil organic matter (and other soil constituents). The strengths of the formed complexes can affect the toxicity and bioavailability of pharmaceuticals, their residues, and their persistence in soils [24,41,42].

In this work, sulphapyridine (as a representative of sulphonamide antibiotics) and diclofenac (as a representative of widely used non-steroidal anti-inflammatory drugs) were chosen for the investigation of their transport in hydrogels. As described above, agarose hydrogels are widely used as a transport medium for different diffusing particles. The enrichment of hydrogels by humic acids can affect the mobility of diffusing particles, as reported in our previous studies [13,14,24,25,26,27]. The effect of humic acids is equivocal because the immobilization of diffusing particles commonly results in a decrease in the diffusion coefficient (suppressing diffusion rate) and (simultaneously) in an increase in the concentration gradient (promoting diffusion rate). It is also necessary to take account of the changes in the viscoelastic properties of hydrogels enriched by humic acids in comparison with pure agarose hydrogels [13,26]. The aim of this study is to investigate the effect of humic acids on the mobility of sulphapyridine and diclofenac, which can provide valuable results utilizable for predicting their behaviour and bioavailability in natural systems (containing organic matter).

## 2. Results and Discussion

**Figure 1:**
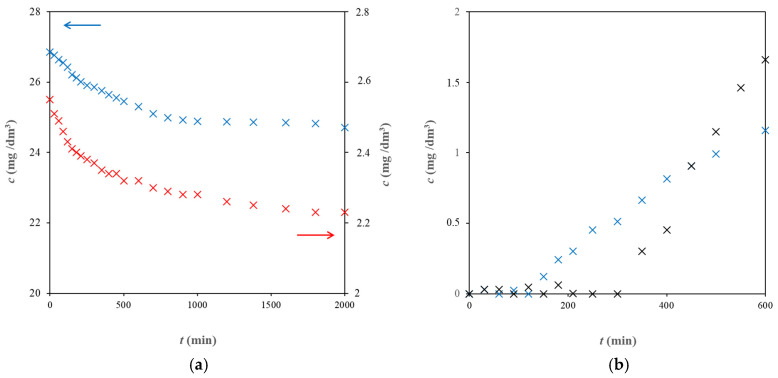
Examples of diffusion data: (**a**) data obtained for sulphapyridine (blue) and diclofenac (red) in the donor part of diffusion cells during the experiment with the pure agarose hydrogel; (**b**) data obtained for sulphapyridine in the acceptor part during the diffusion through the pure agarose hydrogel (blue) and the hydrogel with incorporated humic acids (black).

In Figure 1, examples of experimental data are shown. As can be seen, the concentrations of both studied pharmaceuticals in the donor chambers of diffusion cells decreased gradually in time. The initial concentration of diclofenac was much lower in comparison with sulphapyridine because of the low solubility of diclofenac. The situations in the acceptor parts of diffusion cells were different. In the beginning, the concentrations in the donor chambers were equal to zero, and this state continued up to the so-called lag time [13,26]. This time corresponds to the passing of pharmaceuticals through the hydrogels up to the covering of their thickness between the donor and acceptor parts of diffusion cells. After that, diffusing particles appeared in the acceptor chamber, and the concentration increased. Comparing the experimental data for the pure agarose hydrogel and the hydrogel enriched by humic acids, a prolongation of the lag time for the hydrogel with incorporated active substances was observed. Simultaneously, changes in the slopes of dependencies obtained for the donor chambers were observed around the lag times. This means that the character of the diffusion process changed. In the first phase of the experiment, pharmaceuticals pass through the hydrogel, and their front penetrates into the medium without diffusing particles. If the medium contains an active substance (such as humic acids), diffusing particles can interact with its binding sites and be immobilized in its structure. When diffusing particles pass through the interface between the hydrogel and water in the acceptor chamber, pharmaceuticals appear in it, and the drug concentration increases in this chamber. The lag time (*t_L_*) can be used for the calculation of the diffusion coefficient (*D_L_*) valid for this (first) stage of diffusion [13,26,43,44]:

(1) $$D_{L}=\frac{L^{2}}{6t_{L}}$$

where *L* is the diffusion length (thickness of the hydrogel in this case). The results determined for both studied pharmaceuticals are listed in Table 1.

As can be seen, the lag times determined for sulphapyridine and diclofenac are different. Although the molecular weight of diclofenac is slightly higher than that of sulphapyridine, its lag times of transport through hydrogels are much higher. Thus, the diffusion coefficients (*D_L_*) calculated for diclofenac using Equation (1) are much lower than the values determined for sulphapyridine. The difference is noticeable mainly in the case of the diffusion through the pure agarose hydrogel (AG), where the diffusivity is more than twice as high for sulphapyridine (than that for diclofenac). The lag time is inversely proportional to the diffusion rate in the first stage of the diffusion experiment, which means that a longer lag time shows a lower rate. The diffusion rate is included in the first Fick law

(2) $$j=-D\frac{dc}{dx}$$

where *j* is the diffusion flux, *D* is the diffusion coefficient (the law has general validity; therefore, *D* has no subscript “*L*”), and *c* is the concentration of diffusing particles. The diffusion flux *j* is the amount of a substance diffused through a unit area per a unit of time, and it is proportional to the diffusion coefficient and concentration gradient. Therefore, the diffusion rate is also dependent on the concentration of diffusing particles, the rate of transfer of a diffusant through a unit area is proportional to the gradient of concentration, and the constant of proportionality is the diffusion coefficient [45]. This means that the diffusion rate is dependent on two parameters: the diffusion coefficient and the concentration of diffusing particles (or, more precisely, the concentration distribution). Thus, particles with a higher diffusion coefficient can diffuse at a lower rate due to a low concentration gradient.

If a hydrogel contains an active substance (such as humic acid), diffusing particles can interact with its binding sites while they pass through the hydrogel. The interactions can result in the immobilization of diffusing particles, which stay located in the sites and lose their ability to diffuse. In general, diffusion is usually much slower than chemical reactions; therefore, we can assume that a local equilibrium between immobilized and free movable particles can be quickly established. The equilibrium can be characterized by an apparent equilibrium constant *K*:

(3) $$K=\frac{c_{i}}{c}$$

which is the ratio between the concentration of immobilized (*c_i_*) and free movable particles (*c*). It is necessary to note that the concentration c in the Fick law is also the concentration of free movable particles. This means that the other effect of immobilization is an increase in the concentration gradient (for movable diffusing particles) [13,14,24,25,44].

The apparent equilibrium constant can be included in the diffusion coefficient characterizing transport in the reactive hydrogel (enriched by active substances), as described previously [14,24,25,44]:

(4) $$D_{AG+HA}=\frac{D_{AG}}{1+K}$$

where *D_AG_* and *D_AG+HA_* are the diffusion coefficients of diffusing particles in the pure agarose hydrogel and in the hydrogel enriched by humic acids, respectively. The apparent constant *K* is not the real equilibrium constant defined in thermodynamics because it includes only diffusing and immobilized particles without binding sites of the active substances incorporated in the hydrogel (and potential side products of the reaction eliminated from binding sites). However, the ratio between the diffusion coefficients determined for the inert hydrogel (AG) and the hydrogel with incorporated active substances (AG + HA) can be considered as a criterion of the reactivity and immobilization of pharmaceuticals in enriched hydrogels.

When particles pass through the hydrogel and appear in the acceptor change, the circumstances of diffusion in the hydrogel change. One change is that the particles diffuse through a “saturated” hydrogel, which can suppress their diffusion. While the front of diffusing particles penetrates into the hydrogel without drugs in the first stage of the experiments, the hydrogel pores are filled by the drug solution of a given concentration in the second stage. In the case of the reactive hydrogel (with incorporated active substances), diffusing particles pass through an “equilibrated” hydrogel, which means that the total amount of pharmaceuticals in the hydrogel does not change with time.

The transport of pharmaceuticals can be characterized by an effective diffusion coefficient (*D_E_*), which can be determined on the basis of the Fick law. The concentration gradient (*dc*/*dx*) in Equation (2) was replaced by Δ*c*/Δ*x* = Δ*c*/*L*. Since the concentration of drugs decreased in the donor chamber and increased in the acceptor chamber, the diffusion flux (*j*) and concentration gradient (Δ*c*/*L*) were calculated for each record during measurement. The diffusion coefficient was thus calculated as

(5) $$D_{E}=-\frac{j\cdot L}{∆c}\;$$

where Δ*c* is the difference between concentrations in the acceptor and donor chambers (with respect to the minus sign in the Fick law characterizing the direction of diffusion).

The diffusion coefficients determined for both stages of the diffusion experiments (*D_L_* and *D_E_*) are listed in Table 1. The highest values were obtained for sulphapyridine in the inert hydrogel, and the lowest ones were obtained for diclofenac in the reactive hydrogel with incorporated humic acids. If we compare the obtained results with diffusion coefficients published previously, we can state that their values are strongly affected by the methods of their determinations. In our previous study [24], methods of diffusion from a constant source and the method of the diffusion couple were used. One of the studied pharmaceuticals was sulphapyridine; therefore, we can compare the results for different methods for this drug. While the methods of a constant source and diffusion cells (the second stage of the experiment) gave similar values for the inert agarose hydrogel (4.60 × 10^−10^ and 4.93 × 10^−10^ m^2^/s, respectively) comparable with the value published for the agarose hydrogel used in the passive samplers for in situ measurements (4.75 × 10^−10^ m^2^/s) [46], the value obtained for the diffusion couple was much lower (1.01 × 10^−10^ m^2^/s) [46]. In the case of the reactive hydrogel with incorporated humic acids, the values for sulphapyridine were equal to 3.40 × 10^−10^ m^2^/s (for the constant source) [46] and 9.45 × 10^−11^ m^2^/s (for the diffusion couple) [46]. The diffusion coefficients obtained using diffusion cells in the first stage of the experiment were 5.20 × 10^−10^ m^2^/s (inert hydrogel) and 3.40 × 10^−10^ m^2^/s (reactive hydrogel).

The diffusion coefficients for diclofenac in agarose hydrogels are unobtainable; therefore, our results can be compared only with the diffusion of diclofenac in other systems. Pigall et al. [47] determined the coefficient of the self-diffusion of diclofenac sodium in hydroxypropyl methylcellulose to be between 3.7 × 10^−10^ and 4.0 × 10^−10^ m^2^/s (in dependence on the molar ratio), which is close to the value of *D_L_* in the hydrogel enriched by humic acids. Almbrok et al. [48] determined a value equal to 4.18 × 10^−10^ m^2^/s for the diffusion of diclofenac sodium across the water/1,6-dichlorohexane interface, and Parsee et al. [49] determined diffusion coefficients of the drug from different formulations in the following order: diclofenac lotion (5.31 × 10^−11^ m^2^/s) > lipogel (2.10 × 10^−11^ m^2^/s) > Voltaren emulgel (1.52 × 10^−11^ m^2^/s) > aqueous gel mixed micelle (9.66 × 10^−12^ m^2^/s). Excepting diclofenac formulations [49], the published diffusion coefficients are of the same magnitude as our results. Similarly, the lag time measured for the penetration of diclofenac through rate skin (4.8 h) [50] was close to the lag time measured in the inert agarose hydrogel in our study (5.4 h).

Comparing the results obtained for both pharmaceuticals, we can state that the diffusion coefficients obtained for diclofenac are generally lower than the values determined for sulphapyridine. One reason could be the difference in the molecular weights (and particle sizes) of both pharmaceuticals. Diclofenac has a molecular weight higher by ~20% (in comparison with sulphapyridine), but the decrease in its diffusion coefficients is more considerable. Therefore, differences in particle size can contribute to the decrease in diffusion coefficients, but the main reason is probably more complex. The difference between values of *D_E_* and *D_L_* is higher for diclofenac, which indicates the higher importance of the saturation of inert hydrogels by diffusing particles. Differences between *D_L_* and *D_E_* are connected to the character of diffusion in individual stages of the experiment and can also be shown on a concentration dependence of diffusion coefficients. In the case of the inert agarose hydrogel, pharmaceuticals pass through the hydrogel without interactions, and the difference between both stages of the experiment is in the change in the diffusion character (pure and saturated hydrogels) and the potential effect of the concentration dependence of diffusion coefficient.

If a hydrogel contains an active substance (such as humic acid), the change is also in the “character” of the reaction. This begins in the first stage, and the equilibrium is gradually established as the pharmaceuticals pass through the hydrogel; the equilibrium is preserved in the second stage when new particles pass through the given (equilibrated) place. This means that the equilibrium is dynamic, the concentration of the drug changes at a given place in time, and a new local equilibrium is continually re-established, although the rate of immobilization and liberation of pharmaceuticals are (approximately) the same.

As can be seen, the values of *D_L_* and *D_E_* for both studied pharmaceuticals are lower in the cases of the hydrogels enriched by humic acids. This confirms that the simple model of the local equilibrium between the immobilized and free pharmaceuticals can be used, that the interactions are probably much faster than diffusion, and that the system is (at a given place) equilibrated immediately. The ratios between diffusion coefficients in the inert and reactive hydrogels (with humic acids) were used for the calculation of the apparent equilibrium constants K according to Equation (4), which are listed in Table 2. The results showed that the interactions between the drugs and humic acids are more important in the first stage of the experiment, where *K* is higher than 1 for both pharmaceuticals. The reason is that the new equilibrium is established in the first stage because the front of the diffusing particles passes through the hydrogel, in which all active sites are non-occupied, and the pharmaceuticals begin to interact with them. As mentioned above, we assume that the interactions are much faster than diffusion; therefore, a local equilibrium between immobilized and free movable pharmaceuticals is established practically immediately after their contact.

**Figure 2:**
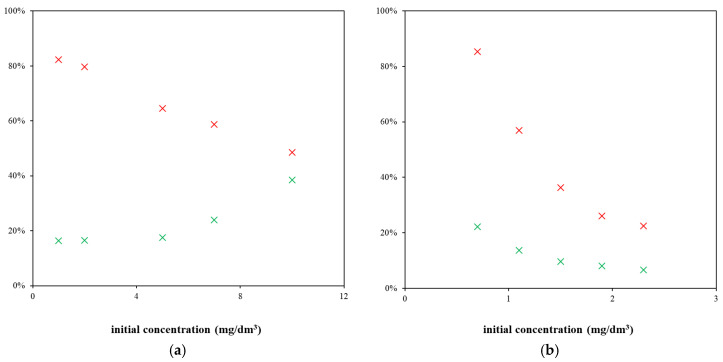
Examples of adsorption/desorption data obtained for sulphapyridine (**a**) and diclofenac (**b**): adsorption efficiency (red) and mobile fraction (green).

In order to investigate interactions between the humic acids and drugs, adsorption and desorption experiments were carried out. As can be seen in Figure 2, the obtained results differ for the studied pharmaceuticals. While the effectiveness of adsorption decreases with increasing concentration for both the studied pharmaceuticals, the desorption experiment showed that the distributions of the strongly bound and mobile fraction of pharmaceuticals are different. In the case of sulphapyridine, the content of mobile particles increases with decreasing adsorption efficiency. This means that a lower adsorbed amount is connected with a higher degree of the liberation of pharmaceuticals. In contrast, the content of the mobile fraction decreases with the adsorption effectiveness in the case of diclofenac. Thus, the situation is the opposite. A similar conclusion can also be indicated from the calculated values of the apparent equilibrium constant *K*, which is close to 1 for diclofenac in the first stage of the experiment where the particles should actively interact and the equilibrium should be just established. The value of *K* calculated for sulphapyridine is practically two times higher; thus, sulphapyridine is much more immobilized in comparison with diclofenac (in this stage). In the second part of the experiment (when the equilibrium is preserved), the values *K* are much lower than 1 for both the studied pharmaceuticals.

It should be noted that observed differences in diffusion coefficients determined for inert and reactive hydrogels can be affected by a little change in the rheological properties of hydrogels caused by the incorporation of humic acids. As described in our previous study [26], humic enrichment shifted the rheological behaviour of a hydrogel to a more liquid character, which can contribute to the decrease in diffusion coefficients.

Another effect influencing transport through a hydrogel layer is the so-called partition coefficient [25,26,27,45,51]. If particles diffuse through a phase interface, a concentration jump can occur at the interface between two different phases. The partition coefficient *ε* can thus be defined as the ratio between concentrations in both phases (at the interface):

(6) $$\varepsilon =-\frac{c_{h}}{c}\;$$

where *c_h_* is the concentration in the hydrogel (and *c* in the solution). The partition coefficient is an equilibrium parameter, which means that the equilibrium is attained across the interface (hydrogel/solution). The coefficient *ε* was calculated based on the mass balance of drugs in diffusion cells. The amount of drug is equal to the difference between the amounts contained in the donor chamber and the acceptor chamber. Due to the equilibrium character of this coefficient, we can use the average concentrations in hydrogels and solutions in both chambers in order to calculate the partition coefficient. Since the hydrogels used in this study contained 99% wt. of water, we assumed that in the case of the inert agarose hydrogel (without humic acids), the partition coefficient should be close to 1. This hypothesis was confirmed by the determination of *ε* (based on experimental data), as shown in Table 2. The increase in the partition coefficient for the reactive hydrogel (with incorporated humic acids) can be attributed to the immobilization of a part of the diffusing particles by the reaction with humic acids. This means that the immobilized particles are included in the concentration of the drug in the hydrogel used for the calculation according to Equation (6). The reason is that we are not able to obtain experimental data directly from the hydrogel, and the drug content of the hydrogel can be determined only based on the difference between the donor and acceptor parts of diffusion cells. However, the partition coefficients determined for both the studied pharmaceuticals are similar, and (therefore) their effect on the assessment of differences between the behaviours of both pharmaceuticals is minor.

## 3. Materials and Methods

### 3.1. Chemicals

Sulphapyridine, diclofenac (sodium salt), and agarose (routine use class) were purchased from Sigma-Aldrich Saint Luis, MO, USA. Elliot soil humic acid standard (1S102H) was purchased from the International Humic Substances Society. The main characteristics of the humic sample can be found on the website of the International Humic Substances Society (http://humic-substances.org/, accessed 1 December 2024).

The preparation of hydrogels was based on the thermo-reversible gelation of agarose aqueous solutions. Agarose was dissolved in deionized water or in an aqueous solution of humic acid, which was heated (80 °C) and stirred to obtain a transparent solution and finally sonicated to remove gases [13,14,24,25,26,27]. Afterwards, the solution was poured into a plastic ring mould fixed between two glass slides. The solution solidified gradually to hydrogel form by spontaneous cooling (to laboratory temperature). When the glasses were removed, a cylindrical hydrogel sample (40 mm in diameter and 5 mm thick) fixed in the plastic mould was obtained [13,26]. Agarose hydrogels were prepared using 1% wt. agarose solution. Enriched hydrogels were prepared from a 1% wt. agarose solution containing 0.01% wt. of humic acid [13,14,24,25,26,27].

### 3.2. Diffusion Experiments

The method of horizontal diffusion cells was used in this work. The used apparatus was purchased from Permegear Inc., Hellertown, U.S. (for details, see our previous studies [13,26]). A hydrogel sample fixed in the plastic mould was placed between two apparatus chambers. The donor chamber was filled with aqueous solutions of a given pharmaceutical, and the acceptor compartment was filled with deionized water; thus, one circular surface of the hydrogel was in contact with the donor solution and the second with water. A circulating water bath was used to maintain the temperature at 25 °C (a scheme of the apparatus and experimental arrangement was published in our previous study [26]). The initial concentrations of pharmaceuticals were equal to 25 mg dm^−3^ for sulphapyridine and 2.5 mg dm^−3^ for diclofenac (because of its lower solubility). The solutions in both compartments were stirred continuously (250 rpm), and the changes in concentrations were monitored by UV/VIS spectrometry (Hitachi U-3900H, Tokyo, Japan). Absorbances at wavelengths equal to 275 nm (diclofenac) and 312 nm (sulphapyridine) were used for the monitoring of concentration changes in diffusion cells.

### 3.3. Adsorption and Desorption Experiments

Adsorption/desorption experiments were realised to investigate interactions between humic acids and pharmaceuticals in detail. The solutions of pharmaceuticals with initial concentrations of 1–10 mg dm^−3^ (sulphapyridine) and 0.5–2.5 mg dm^−3^ (diclofenac) were mixed with humic acids in the ratio of 5 cm^3^ of the solution to 100 mg of the humic sample and stirred for 48 h (to attain an equilibrium). The suspensions were centrifuged, and the decrease in drug concentration was determined by UV/VIS spectrometry (Hitachi U-3900H, Tokyo, Japan). The solid residues (complexes of humic acids with pharmaceuticals) were mixed with deionized water in the same ratio as in the adsorption experiments, equilibrated (48 h), and centrifuged. The concentrations of drugs in leachates were determined by UV/VIS spectrometry (Hitachi U-3900H, Tokyo, Japan). The pharmaceuticals leached into the water were considered as their free movable fraction and the residue as their strongly bound fraction. Absorbances at wavelengths equal to 275 nm (diclofenac) and 312 nm (sulphapyridine) were used for the monitoring of concentration changes in the adsorption/desorption experiments.

All experiments were triplicated and performed at laboratory temperature (25 ± 1 °C).

## 4. Conclusions

The transport of two widely used pharmaceuticals, sulphapyridine (as a representative of sulphonamide antibiotics) and diclofenac (as a representative of widely used non-steroidal anti-inflammatory drugs), was studied in diffusion cells. The aim was to investigate the effect of the incorporation of humic acids into an inert agarose hydrogel on the transport. It was found that the addition of the active (humic) substance into the hydrogel resulted in a decrease in the diffusion coefficients. The realized experiments can be divided into two stages: penetration of pharmaceuticals into the hydrogel and transport through the hydrogel (which is saturated and equilibrated). The incorporation of humic acids into the hydrogel affected the transport into the hydrogel, mainly in the first stage of the experiment when an equilibrium was gradually attained as the pharmaceuticals penetrated into the “pure hydrogel” and contacted the non-occupied humic binding sites. The apparent equilibrium constants determined as the ratio between the concentrations of immobilized (by humic acids) and free movable particles in the hydrogels showed a higher degree of immobilization for sulphapyridine. In the second stage of the experiment, the apparent equilibrium constant *K* was a little higher for diclofenac. The partition coefficient was close to 1 in the case of the inert hydrogel and a little higher than 1 for the reactive hydrogel with incorporated humic acids. The hydrogels contained 99% wt. of water; therefore, the difference in partitioning between the hydrogel and solution phases was minor.

The results obtained in this study provided diffusion coefficients of two widely used pharmaceuticals (sulphapyridine and diclofenac) for their transport in an inert hydrogel and a hydrogel containing humic acids as active substances able to interact with pharmaceuticals during their diffusion. The hydrogel can be considered as a model of a natural soil system with a porous structure and organic matter interacting with diffusing particles and affecting their transport and bioavailability. We believe that the realized experiments and obtained results can provide rewarding data and experiences for the assessment and prediction of the behaviour of pharmaceuticals in nature and their transport properties, bioavailability, and (partial) immobilization by interactions with soil organic matter. Therefore, our research in this field will continue with other pharmaceuticals detected in nature. Simultaneously, we are focused on the influences of different humic substances (extracted from different sources) and other aspects of interactions between pharmaceuticals and organic matter to understand their character.

## Figures and Tables

**Table 1:** Time lag and diffusion coefficients determined for the pure agarose hydrogel (AG) and the hydrogel enriched by humic acids (AG + HA).

Hydrogel Sample	Sulphapyridine			Diclofenac		
	*t_L_* (min)	*D_L_* (10^−10^ m^2^·s^−1^)	*D_E_* (10^−10^ m^2^·s^−1^)	*t_L_* (min)	*D_L_* (10^−10^ m^2^·s^−1^)	*D_E_* (10^−10^ m^2^·s^−1^)
AG	134 ± 4	5.20 ± 0.15	4.93 ± 0.38	323 ± 12	2.34 ± 0.09	1.86 ± 0.05
AG-HA	390 ± 6	1.78 ± 0.03	4.49 ± 0.12	606 ± 27	1.15 ± 0.05	1.46 ± 0.12

**Table 2:** Partition coefficients (*ε*) and apparent equilibrium constants *K*.

Hydrogel Sample	Sulphapyridine			Diclofenac		
	*ε*	*K* First Stage	*K* Second Stage	*ε*	*K* First Stage	*K* Second Stage
AG	0.97 ± 0.05	–	–	1.00 ± 0.01	–	–
AG-HA	1.17 ± 0.02	1.92 ± 0.05	0.10 ± 0.01	1.14 ± 0.01	1.03 ± 0.04	0.27 ± 0.01

## Data Availability

Data will be available on request.
